# Biodegradable Polymers in Bone Tissue Engineering

**DOI:** 10.3390/ma2030833

**Published:** 2009-07-24

**Authors:** Robert J. Kroeze, Marco N. Helder, Leon E. Govaert, Theo H. Smit

**Affiliations:** 1 Department of Orthopaedic Surgery, VU University Medical Center, Amsterdam, The Netherlands; E-Mail: r.kroeze@vumc.nl (R.K.); m.helder@vumc.nl (M.H.); 2 Department of Oral Cell Biology, Academic Centre for Dentistry Amsterdam (ACTA), Universiteit van Amsterdam and Vrije Universiteit, Amsterdam, The Netherlands; 3 MOVE/Skeletal Tissue Engineering Group Amsterdam (STEGA), Amsterdam, The Netherlands; 4 Faculty of Mechanical Engineering, Polymer Technology, Eindhoven University of Technology, Eindhoven, The Netherlands; E-Mail: l.e.govaert@tue.nl (L.G.)

**Keywords:** degradable polymer, tissue engineering, mechanical loading

## Abstract

The use of degradable polymers in medicine largely started around the mid 20^th^ century with their initial use as *in vivo* resorbing sutures. Thorough knowledge on this topic as been gained since then and the potential applications for these polymers were, and still are, rapidly expanding. After improving the properties of lactic acid-based polymers, these were no longer studied only from a scientific point of view, but also for their use in bone surgery in the 1990s. Unfortunately, after implanting these polymers, different foreign body reactions ranging from the presence of white blood cells to sterile sinuses with resorption of the original tissue were observed. This led to the misconception that degradable polymers would, in all cases, lead to inflammation and/or osteolysis at the implantation site. Nowadays, we have accumulated substantial knowledge on the issue of biocompatibility of biodegradable polymers and are able to tailor these polymers for specific applications and thereby strongly reduce the occurrence of adverse tissue reactions. However, the major issue of biofunctionality, when mechanical adaptation is taken into account, has hitherto been largely unrecognized. A thorough understanding of how to improve the biofunctionality, comprising biomechanical stability, but also visualization and sterilization of the material, together with the avoidance of fibrotic tissue formation and foreign body reactions, may greatly enhance the applicability and safety of degradable polymers in a wide area of tissue engineering applications. This review will address our current understanding of these biofunctionality factors, and will subsequently discuss the pitfalls remaining and potential solutions to solve these problems.

## 1. Introduction

Since the first described bone grafting procedure in 1668, when a cranial defect from an injured soldier was successfully repaired using a dog’s skull, considerable improvements have been made [1]. In addition to these improvements, the demand for bone grafting procedures is ever increasing, and the variety of biomaterials available to substitute bone grafts is rapidly expanding. This increased demand reflects the expanded surgical procedures for skeletal reconstructions (e.g. trauma, tumor excision, failed arthroplasty and spinal fusion) resulting in over 2 million grafting procedures annually worldwide [2].

Autografting with bone, often obtained from the iliac crest, remains the “gold standard” since it possesses all the characteristics necessary for new bone growth, i.e. (i) osteoconductivity (passive scaffold to promote vascular ingrowth and bone apposition [3]), (ii) osteogenicity (containing osteoprogenitor cells [3]) and (iii) osteoinductivity (provide signals to induce osteogenic differentiations of local stem cells [3]). However, the use of autografts has its limitations, including predominantly donor-site morbidity (e.g. pain, hematoma, fracture and infection, but also, in particular in children, limited supply), hindering this as an option for bone repair [4,5,6,7,8,9,10,11]. When using donor bone (allografts) other disadvantages have been reported, including transmission of serious diseases from donor to recipient, such as HIV [12] and lymphoma [13], as well as the risk of immune rejection [14]. To circumvent these issues, bone grafting substitutes have become increasingly popular. The basic concept is that the substitute biomaterial acts as a scaffold for the surrounding cells/tissue to invade, grow, and thus guide tissue regeneration towards new bone formation [15,16,17,18,19].

Essentially, biomaterials are designed to promote the organization, growth, and differentiation of cells in the process of forming functional tissue by providing structural support, biological containment, and chemical clues [20]. Advances in cellular biology and material technology, the cornerstones of tissue engineering, are increasingly influencing the clinical practice of various disciplines including orthopedic surgery.

The primary function of skeletal tissues is mechanical support. When a skeletal disorder or tissue damage occurs, fixation is required to reposition the structures involved and to create the proper mechanical environment for functional healing. Once healing is achieved, however, removal is desirable both from a clinical and a biomechanical point of view. Therefore, degradable polymers are increasingly being used in tissue engineering, since they can be used as an implant and will not require a second surgical event for removal [21,22].

In 1994, Freed *et al*. formulated six design criteria for biodegradable polymers which should be met in tissue engineering: (1) the surface should permit cell adhesion and growth; (2) after implantation *in vivo* the polymer and its degradation products should not elicit inflammation or toxicity; (3) the polymer should be reproducibly processable into three dimensional structures; (4) high porosity for reducing diffusion constraints, and increasing surface area and adequate space for extracellular matrix regeneration; (5) the scaffold should resorb after fulfilling its purpose (since foreign materials always carry a risk of inflammation); and lastly (6) the degradation rate of the scaffold should match the rate of tissue regeneration by the cell type of interest [16].

In bone tissue engineering not only do the interactions between degradable polymers and living cells play an important role, but also the interactions between these polymers and the amount and duration of mechanical loading it is required to support. Therefore, optimal interaction both on a cellular level as well as on the biomechanical level is required for a positive outcome in the formation of functional tissue.

Some excellent reviews have discussed the various types of degradable polymers and their co-polymers [22,23,24,25,26,27,28,29]. Therefore, this subject will not be discussed in detail in this review. The scope of this paper is to give a perspective of the facets that enter into (bone) tissue engineering using degradable polymers in particular. Recently, the characteristics of a degradable polymer to be respected prior to implantation have been divided into two main categories: biocompatibility and biofunctionality [30]. Biocompatibility refers to the aspects concerning the absence of toxicity, immunogenicity, carcinogenicity, and thrombogenicity [30]. Biofunctionality refers to the aspects of adequate properties (mechanical, physical, chemical, thermal and biological), easy to handle, sterilizable, storable and resorbable [30].

In order to allow translation of the polymer properties to (human) tissue engineering purposes, a list of commonly used terms in polymer science will be provided following this paragraph. Subsequently degradable polymers will be addressed in two sections. The first will focus on the biocompatibility of degradable polymers, subdivided in foreign body response, surface characteristics and the influence of sterilization thereof. The second section will discuss the biofunctionality issue through visualization *in vivo*, mechanical considerations and the importance of proper mechanical testing. Finally, concluding remarks intending to enhance insight into the requirements needed, not only for the polymers but also for the various scientific fields are provided, since tissue engineering is a broad scientific field, ranging from cells, via biomaterials to practical solutions for treatment strategies in the human body.

## 2. Resorbable Polymer Properties and Nomenclature

Throughout this review, degradable polymers will also be referred to as polymers, to avoid excessive repetition. When non-degradable polymers are discussed, they will be specifically addressed as non-degradable.

Polymeric scaffolds comprise two groups. The first is the group of natural occurring polymers such as polysaccharides (starch, alginate, hyaluronic acid) or proteins (collagen, fibrin, silk), but this review will focus on the second group, the synthetic bioresorbable polymers, such as the poly(α-hydroxy esters).

### 2.1. Molecular weight (M_n_) and intrinsic viscosity

Polymers are large covalent-bound chains, which typically have molecular weights between 10,000 and 1,000,000 [31]. Since not all chains in a polymer are equally sized, a certain distribution of chain lengths is present in the polymer. Therefore, molecular weight is commonly represented as an average value. The most commonly used is the number average molecular weight (M_n_), which is defined as the total weight of all the polymer molecules in a sample is divided by the total number of polymer molecules.

Another way to identify the molecular weight of a polymer is to analyze its intrinsic viscosity IV. When a defined amount of polymer is dissolved to a known concentration and allowed to run through a capillary at a pre-set temperature under gravity, then the transit time can be recorded and compared with that for the pure solvent, allowing the calculation for IV [32]. Intrinsic viscosity thus relates to the ability of a polymer to increase the viscosity of a certain solvent at a given temperature [33].

### 2.2. Crystallinity

**Figure 1:**
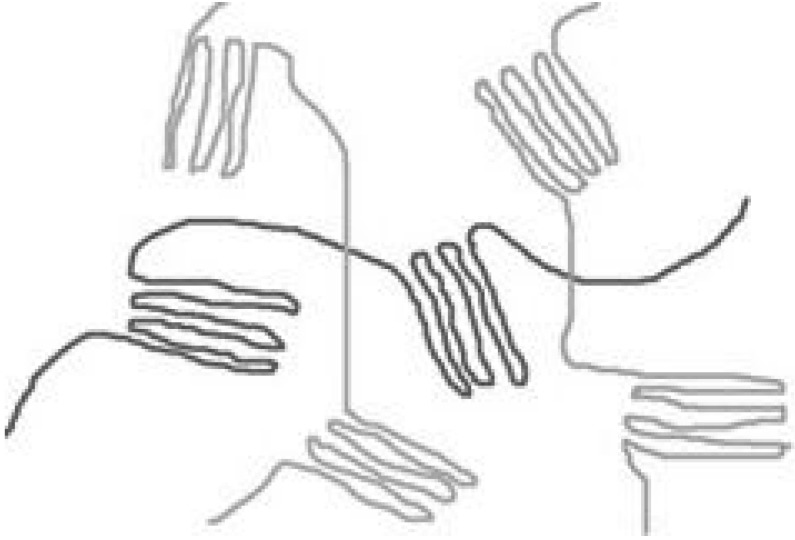
A simplified model of a semi-crystalline polymer. Crystallization stops as non-crystallised polymer length is stretched to the point that further chain movement is impaired. In a three dimensional polymer, this would mean "stretched out", not in a line, but in a random path of "lines" as it bends around neighbouring polymer chains, forming entanglements.

In general, polymers exist either as amorphous or semi-crystalline materials. Amorphous polymers are composed of randomly configured chains, with no long-range order (see Figure 1). Semi-crystalline polymers are heterogeneous systems comprised of highly anisotropic crystallites, a phase in which the chains show long-range 3D order (see Figure 1). The size and distribution of these crystals are extremely dependent on the molecular weight distribution and the conditions under which the material is processed. The prime factor determining whether a polymer can crystallize, is chain regularity since this facilitates regular stacking. The presence of more crystalline regions in a polymer (e.g. higher percentage of crystallinity), will result in improved mechanical properties, combined with slower total degradation time [34]. As crystallinization starts at multiple locations within the scaffold, a polymer is never fully crystalline. PLLA for example, has a maximum crystallinity of around 40%.

### 2.3. Thermal application range

Both amorphous and semi-crystalline polymers have a limited temperature-related application range. The melting point (T_m_) of a highly crystalline (usually non-degradable) polymer implies the transition from molecular order to disorder, or from crystalline to amorphous. Consequently, the T_m_ has limited value in amorphous and semi-crystalline polymers. The glass transition temperature (T_g_) is therefore commonly used and is defined as the onset temperature of main chain segmental motion allowing the chains to change conformation during load. In other words; below the T_g_, the molecular motion effectively ceases [35]. This results in a drastic change in stiffness (modulus) for amorphous polymers, typically from 1 GPa to 1 MPa. In contrast, the mechanical properties of semi-crystalline polymers above T_g_ are fairly preserved with elastic moduli decreasing from their original value of 2.5–4 GPA to 500–1500 MPa. T_g_ can vary drastically among the various types of polymers, e.g. the T_g_ is -60 °C for polycaprolactone but 60 °C for poly(l-lactide) [21].

### 2.4. Co-polymers

All the above mentioned properties or characteristics can change when different monomers are co-polymerized. These co-polymers typically take advantage of the best properties of each of the individual polymers. Depending on the type of co-polymerization used, combining two semi-crystalline polymers can result in either a highly amorphous co-polymer or, due to the presence of homopolymeric blocks inside the co-polymer, a semi-crystalline polymer. The possibility of the material to be ordered into regular patterns is the causal factor in this process. Importantly, molecular weight, T_g_, crystallinity and degradation rate can all be altered by combining different polymers [21,29,34] depending on the type of co-polymerization used.

### 2.5. Degradation

Degradation and erosion are not clearly distinct terms in the literature and some attempt at clarification has been made by claiming that degradation as a chain-scission process whereby polymers degrade to oligomers and finally to monomers, whereas erosion is the process of losing material due to oligomers and monomers leaving the polymer [36,37]. There are various types of polymer degradation such as photo-, thermal-, mechanical-, and chemical degradation [38]. A common denominator for all (non) resorbable polymers is their sensitivity to UV-light and γ-radiation with respect to erosion [38].

An increase in molecular weight will result in more covalent bonds and thus an increased number of entanglements, and thereby increasing resorption/degradation time [34]. The chemical nature of repeating monomers forming the polymer determine the sensitivity of hydrolysable bonds [37,39]. All resorbable polymers contain hydrolysable bonds which are subjected to chemical degradation via either passive hydrolysis or enzyme-catalysed active hydrolysis [39]. For most synthetic polymers, passive hydrolysis is the most important mode of degradation [38,39]. The process of degradation generally occurs through surface erosion and/or bulk erosion. Surface erosion occurs on the surface of the polymer and is related to the impossibility of water to penetrate the polymer in large concentrations. This leads to decreased external dimensions of the implant. All polymers are subject to this type of erosion, but particularly when the implant has a small pore size or a high hydrophobicity [32,38]. Passive hydrolysis is more active in amorphous polymers, or in the amorphous regions in semi-crystalline polymers than in the crystalline regions. This implies that the amorphous region of a polymer degrades faster, leaving the crystalline part to constitute most of the polymer over time. Bulk erosion occurs when water penetrates the implant and breaks the hydrolysable bonds in the backbone of the polymer. In addition to these types of degradation, a process known as autocatalysis can occur, when acids (e.g. lactic or glycolic) are formed and thus lower the local pH during degradation [37,38,39]. When acid production exceeds metabolism, this increased acidity leads to accelerated degradation of the implant [34]. Which way a polymer matrix erodes or degrades depends mainly on: (I) the chemical structure of the polymer (*i.e.*: the covalent bonds that make up the backbone); (II) the intrusion of water inside the matrix; (III) the local pH; and (IV) the dimensions of the matrix [28,37,38].

Finally, the indiscriminate use of the terms biodegradable, bioabsorbable and bioresorbable in literature cause considerable confusion, due to the absence of standard terminology [27]. The term biodegradable can be used for materials that break down *in vivo*, but are not proven to be eliminated from the body, or when the mechanism of degradation is unknown [27,39]. Bioresorbable and bioabsorbable should be used when the polymer is eliminated from the body via either metabolization (to CO_2_ and H_2_O) or because the oligomers are degraded to a size allowing excretion via the kidney [27].

## 3. Biocompatibility

Biocompatibility of a material refers to “the ability of a material to perform with an appropriate host response in a specific situation” [40]. It involves not only the material used, but also the surrounding cells/tissue. The interaction of biomaterials and cells is very complex, and only partially understood [41]. To understand the possibilities to orchestrate the biomaterial-cell reactions, a elucidation of their interactions is needed.

For the allowance of initially cell-free polymers to elicit infiltration of cells, this interaction is of pivotal importance. Under physiological conditions, cells will, amongst others, bind to the surrounding extracellular matrix via ligands. Many proteins interact with cells and thereby evoke a myriad of responses [42]. Since the recognition of biomaterials by a cell is typically mediated by proteins [19,43,44,45,46], preadsorption of specific proteins (or small peptides such as Arg-Gly-Asp; RGD) has been investigated to improve cellular response [43,47,48]. In general, enhancing the biocompatibility of a biomaterial can be achieved by altering the surface characteristics of the substrate, which in turn can lead to enhancing or reducing protein adsorption [43].

### 3.1. Foreign body reaction and fibrous tissue formation

Foreign body reaction is defined as a “response of a host to the presence of a foreign body” [49]. Furthermore it is explained that this reaction is “neither a single event nor a simple process but a broad concept and a multifactorial phenomenon” [49]. This cascade starts chronologically with the injury/implantation, followed by blood-material interactions. This interaction initiates blood protein deposition on the polymer surface, which is described as a provisional matrix. Recently, Wilson *et al*., reviewed this biomaterial-cell interactions via adsorbed proteins [47]. This matrix provides the structural, cellular and biochemical cues for either the wound healing process or the foreign body reaction (FBR). These reactions are for the most part identical, but distinguished by the presence of a foreign body [50]. Anderson *et al*., provides a thorough overview of foreign body reactions to biomaterials [51]. Briefly, in the case of FBR, the acute and chronic inflammatory responses are to follow successively. The chronic FBR for resorbable materials will resolve after full degradation [52].

Since the surface properties can determine the provisional matrix present on the polymer, which consequently can determine the extent or the FBR, the importance of surface properties and protein adsorption has been recognized. Especially the inflammatory cell population, monocytes/macrophages and foreign body giant cells (FBGC; which are fused macrophages and occur when particles are too large for phagocytosis [52]) are key players, dictated by the provisional matrix (for excellent overviews see Refs. [51,52,53]), In this perspective the wettability of a surface can positively increase the biocompatibility *in vitro*, since hydrophilic (and anionic) surfaces have been shown to demonstrate (i) limited adhesion of macrophages (ii) an increased level of apoptosis in adhered macrophages and (iii) a reduced amount of macrophage fusion into FBGCs [54,55] Lastly, degradation of resorbable polymers, such as l-lactide-based polymers, show a non-linear loss of mass, resulting in a increased release of acidic components, possible leading to inflammatory reactions, if not metabolized or drained off quickly enough [56]. In addition, certain degradble polymers can exhibit increased crystallinity during storage at room temperature when compared to storage at 4 °C [57], possible increasing the risk for FBR after implantation.

**Figure 2:**
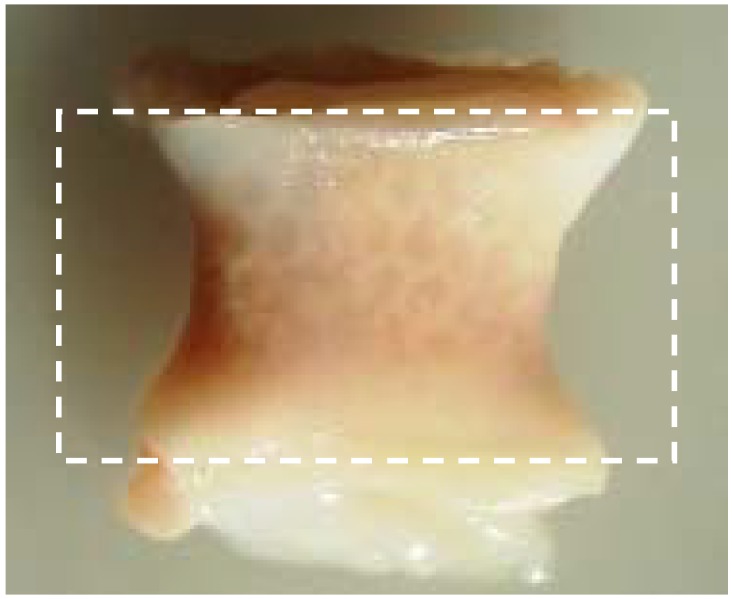
Fibrous encapsulation with clear deformation of the polymer cage filler. The dotted square represents the original size prior to implantation (18 x 10 mm). Retrieval after 6 months.

In some cases granulation tissue precedes the fibrous encapsulation of the implant [51]. Fibrosis can be a major challenge to overcome in tissue engineering. Abundant local production of transforming growth factor-ß (TGF-ß) is the main cause of fibrosis and results in the extensive production of collagens type I and III [52]. Furthermore, TGF-ß initiates differentiation of fibroblast-like cells to myofibroblasts, cells not only critical to wound healing, but also initiators of the formation of fibrotic tissue around the implant [52]. Whether or not the encapsulation occurs and the intensity of the FBR is related to the dimensions of the injury, the biocompatibility of the material, the coverage of provisional matrix and, importantly, the tissue or organ into which the device is implanted [51]. The latter has been established by implanting the polymers PLA and poly(desaminotyrosyl-tyrosine ethyl carbonate) (poly(DTE-carbonate)) in either subcutaneous pouches or in bony defects [58,59,60]. When implanted in a subcutaneous pouch, both polymers exhibited fibrous encapsulation [59], but when implanted into bone, only PLA presented a fibrous tissue lining layer at the bone-implant interface [60]. Moreover, when implanting poly(DTE carbonate) and poly(desaminotyrosyl-tyrosine butyl carbonate) (poly(DTB carbonate)), which have very closely matched chemical structure and material properties, in a bony defect, a dramatic differences in fibrous encapsulation was observed, to the detriment of poly(DTB carbonate) [60]. Animal experiments carried out in our group, in which we implanted a resorbable co-polymer in either bone or in a subcutaneous pouch has shown that the extent of the FBR and the formation of fibrous encapsulation is determined by the anatomical location of the implant site with unfavorable results for the subcutaneous pouch (see Figure 2). Another observation regarding fibrous tissue in all our large animal fusion studies, is the fibrous tissue layer surrounding the outer rim of the spinal cage, with the thickest layer at the load-bearing edges of the cage. Since micro-motion through the spinal motion segment before interbody fusion is unavoidable [61,62], we suspect that the inevitable micro-motion is responsible for the fibrous tissue layer due to cellular shear stress.

For semi-crystalline resorbable polymers, an additional remark has to be made. High crystallinity of the oligomer (low molecular weight fractions that result from degradation and that easily crystallize) can lead to fibrous encapsulation when implanted subcutaneously [59]. Late inflammatory responses can occur, since the amorphous region of a polymer is degraded the earliest [32], leaving the degradation-resistant crystals to invoke a FBR [27,32,63].

### 3.2. Surface characteristics and sterilization

It is known that cells are sensitive to subtle differences in surface characteristics [46] and by analysing the surface characteristics and/or modifications, such as topography, chemistry and hydrophilicity in relation to the behavior of cells, more aspects in cell/biomaterial interaction have been clarified [46,64,65,66,67]. An extensive assessment of stem cell/biomaterials combinations has, for example, been performed by Neuss *et al*. [41]

The topography of the surface of the substrate, which can vary (or can be varied) from macro to micro, can determine the response of cells which are typically 10-100 μm in diameter [42]. For non-resorbable orthopaedic implants, the effect of the surface roughness (often referred to as root mean square (RMS), representing an average of the peaks and valleys of the surface) on the cellular response and on the bone-implant interface is being delineated [46,68,69,70]. In general, increased surface roughness is associated with decreased proliferation and increased differentiation of the cells [69]. More accurately, it is found that the response of the cell is cell-type specific and roughness dependent. It has been shown that osteoblasts and bone marrow cells preferably attach to and proliferate on rougher surfaces, while fibroblasts and epithelial cells prefer smoother and extreme smooth surfaces respectively [46,71,72]. This might indicate cell specific preferences with respect to surface roughness for their tissue of origin. In this respect, Thapa *et al.* recently showed an increased bladder smooth muscle cell adhesion to a resorbable polymer by mimicking the topography of native bladder tissue [73].

Another variable characteristic of the substrate surface is the hydrophilicity or wettability. An increased hydrophilicity of the polymer as a cell substrate leads to increased cell attachment and higher proliferation rates of the cultured cells [74,75]. Furthermore, for bone tissue engineering purposes, it has been stated that an increase in substrate wettability will result in an increased activity of alkaline phosphatase (ALP, indicating osteogenic potential of the cultured cells), not only for osteoblasts [76,77] but also for mesenchymal stem cells [64,78]. However, contradictory results in other studies, showed an inverse relation with ALP activity of cultured cells and the wettability [79,80]. Moreover, the hydrophilicity of a substrate can also affect the host response *in vitro* by altering the cellular reaction of a immune responsive white blood cell, the monocyte. With greater hydrophilicity, not only lower amounts of monocytes attached to the surface but also increased apoptosis (programmed cell death) occured for the adhered monocyte fraction [54,55]. Therefore, the hydrophilicity of the polymer could potentially be used to reduce immune responses *in vivo*.

Irrespective of the biomaterial used, sterilization is mandatory for clinical usage, but the method used can substantially affect the physical/mechanical properties of bioabsorbable polymers [81,82]. The T_g_ of many polymers would be exceeded during hospital steam sterilization and their physical and mechanical properties thus altered, making this technique unsuitable for resorbable polymers [83].

For heat sensitive polymers, ethylene-oxide sterilization can be used. Ethylene-oxide (EtO) gas sterilization is a direct alkylating method and therefore a microbiological inactivator. [84] EtO has been shown to leave the physical and structural properties of the bioabsorbable polymer poly(beta-hydroxy octanoate) unaltered [85]. Since EtO does not involve the radiation by particles, it is more likely to preserve original wettability and surface roughness properties of the bulk polymer.

In contrast, some sterilization techniques and/or sterilizing agents can specifically be used to alter the surface roughness and hydrophilicity of the surface. Simply immersing polymers in alkaloids and/or ethanol can lead to effective improvement of the surface [86,87]. Other common procedures of surface functionalization include plasma treatment or glow discharge capable of increasing the wettability of (resorbable) polymers by creating reactive sites via the interaction of ionized molecules with the polymer [75]. An increased wettability, as achieved by glow discharge, could likewise be induced in our experiments [80] by using clinically applied electron-beam sterilization. Our increased wettability of the polymer after e-beam sterilization is in accordance with increased hydrophilicity of the ceramic hydroxyapatite [88] and various (non-resorbable) polymers [89], due to surface oxidation in a dose dependent manner. Since sterilization of the substrate is mandatory before clinical application, the proper technique should be evaluated, since some techniques, like e-beam, can achieve additional positive outcomes for surface functionalization, while care should be taken since the mechanical properties of the polymer can simultaneously be affected.

Since sterilization can affect not only the mechanical properties, but also the surface properties of polymers, affecting the cell-material interaction, the complete sequence of the production of the polymer, including sterilization, should be evaluated prior to pre-clinical testing.

In summary, the biocompatibility of resorbable implants could be at risk by causing long-term complications, such as local osteolysis, the formation of sterile sinuses and fibrous encapsulation [21,32,90,91,92,93,94,95,96,97,98]. The intensity of this host tissue response is influenced by implant related factors e.g. surface properties, polymer type, purity, crystallinity, design and processing techniques. Since the last decade, many groups were able to show good bio(osteo)compatibility for various polymers for bone tissue engineering *in vivo* [98,99,100,101]. Recently, bioresorbable polymers have been used to correct cranio-facial deformities in a multi-center EU trial [102]. For self-reinforced PLA, Ashammakhi *et al*., reported very low prevalence (0.1%) of clinically manifested inflammatory reactions [100]. A recent overview on the use of polymers in clinical trials has been written by Pietrzak *et al*. [32].

## 4. Biofunctionality

When bony tissues fail due to trauma or disease, additional support is required to take over their mechanical function. For example, in spinal diseases causing degeneration, instability and/or severe deformations, spinal fusion of the segments may be needed. Devices used for this purpose should not only maintain or restore the spinal anatomy, but also create the proper mechanical environment for bony fusion. The load bearing device for interbody spinal fusion is the so-called cage, which usually is supplied with a load-transducing filler material. As bones and implants must resist considerable loads, metals and/or alloys are popular load-bearing materials used for cages.

**Figure 3:**
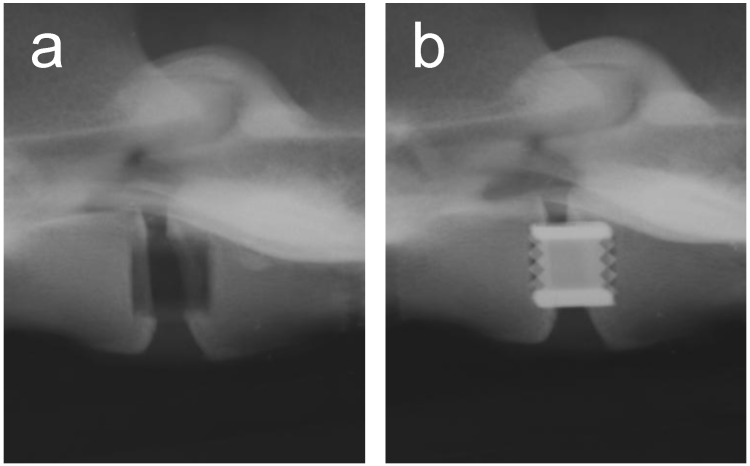
A postoperative lateral roentgenogram of an explanted goat spine with the placement of a radiolucent cage (a) and a titanium cage (b).

**Figure 4:**
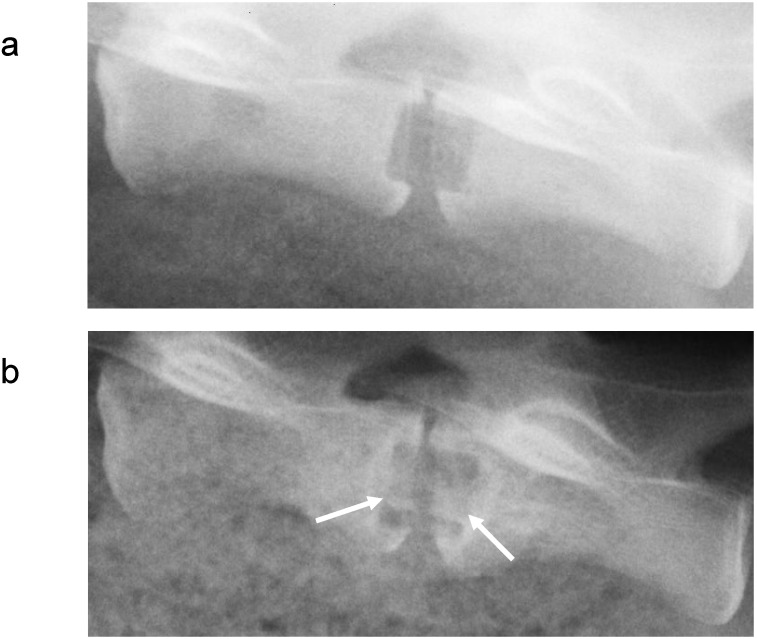
Lateral roentgenogram of a goat, using both a radiolucent cage and polymer cage filler (a) directly postoperative and (b) prior to sacrifice, 6 months after surgery, in which bone formation in the cage, indicated by the white arrows, can be seen.

Metals and/or alloys have proven to be successful, although drawbacks do exist. In spinal surgery, amongst others, permanent materials such as metals (and non-resorbable polymers) remain susceptible to long-term complications such as migration [103], wear [61], late foreign body reaction [61,104] and infection [105]. The inflammatory reaction is, in some cases, the result of the inevitable corrosion of alloys *in vivo* (often referred to as particle disease), and also in the spine [106,107]. In other cases, the aforementioned micro-motion through the spinal motion segment may lead to particle debris [22]. Therefore strategies to minimize implant related problems have been devised such as removal of the implant after fulfilling its purpose in every patient [108], or to selectively remove the implant in symptomatic patients [95], which in return can cause neurovascular injury or refracture [95]. In the USA, retrieval surgeries of the spine were reported in 25-40% of the patients [109,110,111]. Furthermore, metallic spinal implants are strongly radiopaque on roentgenograms, which is the most widely used follow-up imaging after spinal surgery [112] (see Figure 3). This results in an obscured view and therefore hampered assessment of fusion, since the presence of a bony bridge throughout the spinal implant can not be seen [112,113,114,115]. Not only do metals/alloys interfere with simple x-ray films, they will therefore also interfere with computer axial tomography scanning (CAT) and cause artefacts (scattering) with magnetic resonance imaging (MRI) [100] In contrast, the presence of a bony bridge on a plain roentgenogram in radiolucent spinal implants can be visualized and does correlate with surgical exploration, considered the gold standard [116]. Radiolucent spinal implants are generally made from non-degradable polymers such as polyetheretherketone (PEEK) and will also not interfere with CAT scans or MRI scans. However, in a similar fashion as metallic cages, non-degradable cages will remain susceptible to similar long-term complications. Development of degradable spinal cages will not only result in optimal assessment of spinal fusion during follow-up using x-ray films (see Figure 4), CAT scans or MRI scans, but also avert potential long-term complications, resulting in a patient-friendly and cost-effective treatment option.

Finally, when an incommensurate amount of load is directed through the load-bearing implant, the chance of achieving bony fusion decreases and can result in fragile, thin bone trabeculae or non-union [117]. This so-called stress shielding can occur due to the discrepancy between the much higher stiffness of the metallic implant compared to that of vertebral bone [117,118,119,120]. In this respect, a higher stiffness can work contra productive. In our goat spinal fusion studies we found that a ‘flexible’ PLLA cage (axial compression stiffness (ACS): 2 kN/mm) contained significant higher percentage of lamellar bone compared to the ‘stiff’ PLLA cage(ACS:4 kN/mm) after 6 months *in vivo* [121]. In the same study, a higher rate of fusion was detected for the PLLA cages when compared to the titanium cages after 6 months [121].

Mechanical strength should be considered carefully when using resorbable implants, since polymers have limited strength compared to metals. When designing degradable implants it is important to realize the type and magnitude of load the implant is expected to support. An implant can therefore act as a non-load bearing or load-transducing scaffold for cells to grow in, or can function as a load bearing scaffold, expected to maintain mechanical stability and integrity. This dichotomy between non-load bearing and load bearing requires different polymer properties. Usually, glassy degradable polymers are being used for load-bearing properties. Degradable bone screws and plates, made of these glassy polymers, have yielded good results in recent research [32], and despite the fact that the spine is among the most difficult anatomical locations in the body to achieve fusion [32], polymer implants have achieved spinal fusion *in vivo* [32,119,121,122,123,124].

The human skeleton –in particular the spine and long bones- is subjected to relatively large dynamic loading ranges and polymers appear to degrade faster under such conditions [21,125]. Too early loss of mechanical integrity results in instability of the spinal segment and ultimately in non-unions and clinical failure [122]. Moreover, glassy polymers show a strong time- and load-dependent behavior [108]. In our own animal spinal fusion experiments, we have experienced this behavior, since the mechanical strength of the co-polymeric cage, was lower for lower (!) loading rates, higher temperature and higher humidity [126]. Since many materials (e.g metal) can endure any static load below the yield strength almost indefinitely, tests concerning static loading are uncommon and not routinely performed on polymers. Therefore, despite the preliminary compressive mechanical tests according to the American Society for Testing and materials (ASTM) standards (F2077-03) [122], to our surprise, premature failure of the interbody fusion cages was observed within less than 5 minutes (!) when *statically* loaded at 75% of their strength, indicating the need to reconsider the standards for mechanical testing of strongly time-dependent materials [126]. The elucidation of these problems for resorbable polymers is clearly explained by Govaert *et al*. [108] In addition to static loading, the aforementioned ASTM test also requires a perfect fit for interbody fusion devices in the holder during testing. In a clinical setting, a perfect fit is rare, but rather unproblematic for metallic devices. For polymeric cages, the absence of a perfect fit results in concentrations in tensile stress due to three-point bending and subsequent brittle failure. In the animal experiments of Krijnen *et al*., we have experienced advanced mechanical degradation of resorbable lumbar interbody fusion cages due to e-beam sterilization as compared to ethylene-oxide sterilization [82,122]. Smit *et al*., showed that for *in vitro* experiments, e-beam induced a loss of molecular weight by a factor three, when compared to EtO. This equals an EtO sterilized cage after about 11.5 months of degradation [82].

Since living bone is a self-optimizing tissue through mechanical adaptation [118,127,128], bone in itself is the optimal mechanical support. Therefore skeletal devices should essentially have a temporary function and should subsequently be removed both from a clinical and biomechanical point of view, once healing is achieved. Degradable polymers can be tailored to match the stiffness of bone [120], do not interfere with several imaging techniques [129] and degrade over time in which the mechanical load can be gradually transferred from the resorbable implant to newly formed tissue [130] and eliminating the potential need for removal surgery.

Since 1996, our group has pursued this ‘temporary’ skeletal engineering approach for the spine, and it has resulted in the development of a bioabsorbable implant, which is currently in clinical evaluation [22,82,121,126,131,132,133].

## 5. Emerging Technologies

Despite the potential pitfalls in bone tissue engineering when using degradable polymers, many caveats are being delineated and advantages are increasingly being recognized by clinicians. As a result, the market for degradable polymers in surgery continues to grow. When using degradable polymers in surgery for bone repair, its cost-effectiveness has been demonstrated in several studies, not only due to the omission of removal surgery, but also due to the decrease in sick-leave time and the number of radiographs taken [100,134,135]. After implantation, Sinisaari and colleagues detected lower infection rates for degradable fixation devices in ankle surgery when compared to their metallic counterparts [136,137].

More recently, a new generation of implants is emerging with the possibility of incorporating antibiotic substances in the polymers which will be released in a controlled manner during degradation. Implants made of polyglycolide-co-polylactide (PLGA) and PDLA are currently being studied in animal models to assess the risk of infection after implantation [100,136,137,138,139,140,141]. Combining the function of scaffold and controlled release system holds great potential for degradable polymers, not only for antibiotic substances, but also for various growth factors (e.g. vascular endothelial growth factor, bone morphogenetic proteins), thereby possibly shortening the bone healing process [26,31]. Another example of these are aliphatic polyesters like polyhydroxybutyrate (PHB) which are produced by micro-organisms. Their major degradation product, hydroxybutyric acid, is naturally found in human blood. The PHB homopolymer is highly crystalline and brittle; this can be overcome by copolymerizing with polyhydroxyvalerate (PHV) to reduce crystallinity from 80 to 35% and to increase failure strain from 8-50%. Mechanical properties of polymers thus can be improved in order to meet requirements of specific implantation sites.

Degradable scaffolds for bone tissue engineering not only should meet requirements on material properties, but also geometric specifications have to be fulfilled. In general, the higher the porosity and the pore-size of the scaffold, the greater the bone ingrowth *in vivo* [31]. A porosity of at least 80-90% [142,143] and a pore-size of at least 300 μm [144,145] are recommended due to improved bone formation and the formation of capillaries, pivotal for nutrient support. Various technologies are available for that, such as salt-leaching techniques or —for more regular shapes— polymer printing technology.

A novel design approach for scaffolds is biomimetic design. In order to mimic the extracellular matrix (ECM), nanofiber-based scaffolds, prepared by electrospinning, contain fibers in nano-scale range which resemble biological fibers in the extra cellular matrix [146]. Nanofiber based polymeric scaffolds have shown increased cell attachment, and higher alkaline phosphatase activity when compared to matrices without nanofibers [146]. Other biomimetic design approaches include the incorporation of synthesized forms of the calcium phosphates naturally occurring in bone in polymers [26]. These so-called composite scaffolds present several advantages. Firstly, during degradation of the polymer, a lowering of the pH may occur. This increased acidity can be buffered by the calcium phosphate, thus dampening the acidic polymer degradation [26]. Secondly, slight increase in stiffness and strength was found for composites when compared to the polymer alone [26]. Thirdly, by incorporating calcium phosphates, direct bone apposition can occur at the polymer-bone interface [147] (osteoconduction), preventing the unfavorable fibrous encapsulation of the scaffold [60]. In contrast to incorporating calcium phosphates it has recently been shown that after surface functionalization of the polymer via hydrolysis, a chemically bonded calcium phosphate coating (crystalline apatite [148]) can be generated. Cowan *et al*., has shown increased bone formation in mice cranial defects after implanting osteoblast seeded, pre-mineralized PLGA scaffolds when compared to osteoblast seeded, but not pre-mineralized PLGA scaffolds [149].

With the increased interest in minimally invasive surgery, patients experience less pain, reduced scarring and tissue injury, and shortened hospital time. A major disadvantage of this development is that the smaller openings in the patients make it more difficult to insert an implant or to knot a suture. To address this issue, degradable elastic shape memory polymers may be of use, that can take a certain shape upon thermal or optic induction [56,150]. These polymers may be introduced in a compressed or bent form and obtain the appropriate shape upon induction. Various polymers can be used for this purpose, including PCL-co-PLLA (PCLA), PDLLA-hydroxyapatite composites, and copolyester-urethane networks [151,152].

The possibilities of degradable polymers are steadily increasing and in fact are currently overwhelming. Material design traditionally starts with the synthesis of a new material, which is then characterized chemically, biologically and mechanically, and then a suitable application can be identified. Computational modeling may speed up this process by predicting the behavior of polymers under various conditions. Libraries containing thousands of individual polymer compositions can be used to predict material characteristics like elastic modulus, degradation time, and glass-transition temperature as a function of the manufacturing process and implantation time [153,154]. Also the time-dependent properties of polymers under static and dynamic loading conditions can be predicted [108]. Using such databases, design factors like shape, surface topography and chemical composition can be controlled to ensure that implants will meet the appropriate requirements of the specific application. Furthermore, customized implants can be created by freeform fabrication and polymer scaffold libraries [155,156,157,158,159]. Solid freeform fabrication allows for 3D printing of custom designs using CAD drawings. Polymer scaffold libraries allow determining appropriate cell-scaffold combinations that will be successful in tissue engineering applications. Polymer expert systems such as these will prove helpful if not mandatory in designing new implants of degradable polymers and thereby enhance the tissue engineering process.

## 6. Conclusions

The early papers on resorbable polymers demonstrated quite some adverse effects, causing negative publicity and barring of degradable polymers from clinical evaluation. With our increased knowledge of the principle pathways causing these effects, many adverse effects have been clarified and can be avoided. The orchestration of an appropriate host tissue response is not only guided by the biocompatibility (intrinsic) issues of the polymer but also by the biofunctionality issues, which we consider to be the implantation site, the vascularization of the scaffold, the presence/absence of micro-motion, the dynamic loading regime and the visualization *in vivo*. Critical biocompatibility problems, such as fibrous encapsulation has been studied extensively, and can likely be circumvented via direct bone apposition at the surface. Despite the fact that the biofunctionality of degradable polymers such as mechanical loading of polymeric scaffolds requires further investigation as well as adaptation of mechanical testing of materials, pivotal biofunctionality issues are often overlooked.

Since degradable polymers are part of a comprehensive field of research, a multidisciplinary approach is crucial and the convergence of various scientific fields should lead to a cost-effective, patient-friendly strategy to treat bone defects. Once pre-mineralization, growth factor release systems and other biomimetic approaches are clinically available, and have had proper mechanical test runs, degradable polymers will get their second chance in clinical practice. Computational modeling will be helpful in selecting appropriate polymers out of thousands of candidates stored in libraries and predict their properties during manufacturing and their behavior after implantation over the degradation process.
